# Decoding the impact of interspecies interactions on biofilm matrix components

**DOI:** 10.1016/j.bioflm.2025.100271

**Published:** 2025-03-14

**Authors:** Cristina I. Amador, Henriette L. Røder, Jakob Herschend, Thomas R. Neu, Mette Burmølle

**Affiliations:** a Section of Microbiology, Department of Biology, University of Copenhagen, Copenhagen, Denmark; b Department of River Ecology, Helmholtz Centre for Environmental Research - UFZ, Magdeburg, Germany

**Keywords:** Multispecies biofilms, Interspecies interactions, Glycans, Extracellular polymeric substances

## Abstract

Multispecies biofilms are complex communities where extracellular polymeric substances (EPS) shape structure, adaptability, and functionality. However, characterizing the components of EPS, particularly glycans and proteins, remains a challenge due to the diverse bacterial species present and their interactions within the matrix. This study examined how interactions between different species affect EPS component composition and spatial organization. We analyzed a consortium of four bacterial soil isolates that have previously demonstrated various intrinsic properties in biofilm communities: *Microbacterium oxydans*, *Paenibacillus amylolyticus*, *Stenotrophomonas rhizophila*, and *Xanthomonas retroflexus*. We used fluorescence lectin binding analysis to identify specific glycan components and *meta*-proteomics to characterize matrix proteins in mono- and multispecies biofilms. Our results revealed diverse glycan structures and composition, including fucose and different amino sugar-containing polymers, with substantial differences between monospecies and multispecies biofilms. In isolation, *M. oxydans* produced galactose/N-Acetylgalactosamine network-like structures and influenced the matrix composition in multispecies biofilms. Proteomic analysis revealed presence of flagellin proteins in *X. retroflexus* and *P. amylolyticus*, particularly in multispecies biofilms. Additionally, surface-layer proteins and a unique peroxidase were identified in *P. amylolyticus* multispecies biofilms, indicating enhanced oxidative stress resistance and structural stability under these conditions. This study highlights the crucial role of interspecies interactions in shaping the biofilm matrix, as well as the production of glycans and proteins, thereby enhancing our understanding of biofilm complexity.

## 1 Introduction

Biofilms constitute a unique growth mode for microorganisms that allow them to survive in hostile environments. Unlike the planktonic lifestyle, cells in a biofilm arrange as aggregates and are embedded in a matrix of extracellular polymeric substances (EPS) [1,2]. EPS, also referred to as the biofilm matrix, comprises extracellular DNA, lipids, exopolysaccharides, and extracellular proteins. Structural components including flagella, pili, fimbriae, and amyloids can stabilize the matrix, and generally, the biofilm matrix determines the architecture, function, mechanical stability, and dynamics of the EPS matrix in different ways [[3], [4], [5]]. Extracellular polysaccharides and glycoconjugates, which are carbohydrates linked to non-sugar molecules, impact cellular adhesion, cell-cell interactions, biofilm stability, and nutrition [6]. The proteinaceous components of the biofilm matrix include secreted extracellular proteins, cell surface adhesins, and cell appendages such as flagella and pili [3]. Additionally, some matrix proteins serve functional roles through their enzymatic activity, including the degradation of biopolymers, involvement in biochemical processes, or signaling [7]. However, the composition and structure of EPS can vary greatly depending on the type of microorganisms, local shear stress, availability of nutrients/substrates, and the host environment [5].

In nature, biofilms are usually composed of multiple species of bacteria, where interspecies interactions may result in emergent functions and capabilities [8,9]. An example of such intrinsic properties is the multispecies biofilm model composed of *X. retroflexus*, *S. rhizophila*, *M. oxydans*, and *P. amylolyticus* [10,11]. This bacterial consortium shows community-intrinsic properties compared to its members in isolation, including synergistic biofilm biomass [12,10], metabolic cross-feeding [11,13], pH stabilization [14,15], improved degradation of keratin [16], and plant protection and growth promotion [17,18]. Biofilm synergy in this consortium has been validated in different biofilm settings, such as multi-well plates, drip-flow reactors, or root surfaces [[10], [11], [13], [18], [19]]. Remarkably, all species are required for the synergistic effects described.

Community intrinsic properties, however, have often been studied with bacterial cells as the primary subject for analysis, ignoring the biofilm matrix. This leaves the biofilm matrix and its role in facilitating emergent properties underexplored, despite the fact that some studies hint towards matrix component interactions and differential expressions in multispecies settings [20]. As examples, Guillonneau et al. reported induced synthesis of manose-glucose exopolysacharides while Klein et al. described induced expression of glucan synthesis in *Streptococcus mutants* in response to other species [21,22]. Moreover, Yannarell et al. reported that interactions between *Bacillus subtilis* and *Pantoea agglomerans* led to a protective effect towards the latter from antimicrobial killing. Such protection depended on the TasA amyloid, produced by *B. subtilis*, and exopolysaccharides from *P. agglomerans* [23].

Few studies have used proteomics to specifically identify matrix proteins in *Pseudomonas aeruginosa* or *Staphylococcus aureus* biofilms, and primarily at the single species level [7,24]. Others have employed *meta*-proteomics to identify the presence and abundance of proteins in biofilm communities but with a focus on taxonomic characterization or metabolic functionality [[25], [26], [27]]. We have previously demonstrated the potential of *meta*-proteomics for investigating the mechanisms influencing biofilm formation in communities. Herschend et al. proved that the community relied on cooperative interactions among its members, enabling cross-feeding on particular amino acids [13]. However, this was done using total biofilm samples, including proteins from biofilm cells and from the matrix. Other techniques, such as confocal laser scanning microscopy (CLSM) coupled with fluorescent staining, have proven to be the most flexible approach for imaging biofilm EPS, where specific staining, applied to functional groups in polymers, allows identification and localization of different matrix constituents [20,28]. Various studies have utilized fluorescently labeled lectins to analyze and image EPS glycoconjugates in different types of biofilms [[29], [30], [31], [32]].

In this study, we investigated how interspecies interactions influence the production of specific matrix glycans and proteins in multispecies biofilms. We employed the well-studied multispecies biofilm model, composed of *M. oxydans* (MO), *P. amylolyticus* (PA), *S. rhizophila* (SR), and *X. retroflexus* (XR), and combined matrix proteomics with fluorescent lectin staining. The former aimed at enriching extracellular, membrane, or surface associated proteins using a matrix extraction, combined with mass spectrometry, to identify proteins differentially or uniquely detected in mono- *vs*. multispecies biofilms. Moreover, in a microbial community, each species may produce a unique set of glycan molecules, adding to the complexity of deciphering the EPS glycome. Thus, fluorescent lectin staining is aimed at characterizing the identity and spatial organization of glycans in mono- *vs*. multispecies biofilms. We hypothesized that the production of specific matrix proteins and glycoconjugates may be intrinsic to interspecies interactions and, therefore, unpredictable from monospecies biofilm analysis.

## 2 Experimental procedures

### 2.1 Strains and materials

The strains used in this study—MO, PA, SR, and XR—derived from a maize decaying leaf [12,33]*.* All strains were grown at 24 °C in Tryptic Soy Broth (TSB; 17 g pancreatic digest of casein, 3 g papaic digest of soybean meal, 5 g sodium chloride, 2.5 g dextrose, 2.5 g dibasic potassium phosphate in 1 l distilled water, pH 7.3) overnight (>18 h) and 250 rpm. Media was supplemented with 1.5 % agar when needed.

To distinguish the bacterial strains, 40 μg/ml of Congo Red (Merck) and 20 μg/ml Coomassie Brilliant Blue G (Sigma-Aldrich) were added to the TSB base, referred to as TSB Congo Red plates. In previous studies, this media has facilitated the identification of adapted variants and differentiation among species [14,34].

### 2.2 Construction of fluorescently tagged *X. retroflexus*

GFPmut3 was introduced into XR using the mini-Tn7 system following the general procedures described by Choi and Schweizer [35]: Cells were made electrocompetent by centrifuging (2 min, 10,000 g, 4 °C) 1.5 ml overnight culture grown in LB at 30 °C while shaking at 250 rpm. Next, the pellet was washed (2 min, 10000×*g*, 4 °C) three times in 1 ml ice-cold 300 mM sucrose. After a final centrifugation step, the pellet was resuspended in 50 μl 300 mM sucrose. The electrocompetent XR strain was transformed with 25 ng of helper plasmid pTNS2 and 25 ng delivery plasmid pUC18T-miniTn7-PlppGFPmut3-TcR [36]. Electroporation was performed using pre-chilled 1 mm gap electroporation cuvettes (BIO-RAD) and a Micropulser electroporation apparatus (BIO-RAD), followed by recovery in pre-heated 30 °C LB broth for 2 h (h) at 250 rpm. Selection was done on TSB agar plates supplemented with 30 μg/ml tetracycline. PCR and fluorescence microscopy verified the insertion of PlppGFPmut3-TcR (via pUC18T-miniTn7-PlppGFPmut3-TcR) into the chromosome of XR. This strain was used for lectin staining assays, using the lectin RCA-Rhodamine to examine the potential production of matrix glycans.

### 2.3 Cultivation of biofilms

Overnight cultures in TSB medium were adjusted to an optical density at 600 nm (OD_600_) of 0.15 in fresh TSB before usage. The experiment was conducted in 24-well plates, where each well contained a polycarbonate (PC) chip (12 x 12 × 0.78 mm^3^) that was diagonally tilted so bacteria could adhere to both sides of the chip. In each well, 2 ml of OD_600_ 0.15 cultures were added as mono-species or mixed-species cultures. Species ratios of 1:1 or 1:1:1:1 OD600 were used for mixed-species conditions. Multi-well plates were incubated for 24 h at 24 °C in static conditions.

### 2.4 Biofilm matrix staining with fluorescent lectins

Matrix glycoconjugate characterization of multispecies biofilms was performed in a screening using 78 different fluorescently labeled lectins (using FITC, AlexaFluor488, or Fluorescein) in combination with CLSM (TCS SP5X, Leica Germany, controlled by the software program LAS-AF vers. 2.41) as described previously by Neu and Kuhlicke [31,37]. The lectins used, the residues they bind, and fluorescent conjugates used are listed in Supplementary Table S1. Briefly, biofilms were grown on polycarbonate chips for 24 h, followed by washing once with 1x PBS, as described above. Staining solutions containing fluorescent lectins were prepared at a concentration of 100 μg/ml (in filter-sterilized ultrapure water). 100 μl of such lectin solutions were added to a biofilm on a polycarbonate chip, followed by incubation for 30 min in the dark. The excess staining solution was washed twice with dH_2_O before adding SYTO60 to the sample before confocal imaging. FITC, AlexaFluor488, and Fluorescein were excited at 488 nm, and the emission signals were recorded from 500 to 580 nm. Excitation of SYTO60 was at 652 nm, and emission was recorded from 665 to 750 nm. For lectins conjugated to Rhodamine, excitation was done at 525 nm, and emission was recorded from 570 to 650 nm.

Image data sets were recorded using 63x NA 0.9 and 63x NA 1.2 objective lenses with a step size of 0.5 μm, capturing Z-stacks, which involve imaging multiple layers at different focal depths to create a 3D representation. Maximum intensity projection (MIP) images were generated from three-dimensional (3D) data sets using IMARIS version 8.3.1. This process converts the 3D image stack into a two-dimensional (2D) image by selecting the brightest pixel at each position, preserving key structural information from the original dataset.

Based on the screening, four lectins, *Iberis Amara (IAA)*, *Ricinus communis* Agglutinin (RCA), *Vicia Villosa* (VVA), and Wheat Germ Agglutinin (WGA) were chosen for further testing. Here, monospecies and multispecies biofilms were used for fluorescent lectin and SYTO60 staining along with dual species combinations of XR and PA. A minimum of 6 images per sample (≥3 biological replicates and 2 images per replicate) were acquired for each sample type and lectin.

### 2.5 Calculation of biofilm thickness and statistical analysis

Biofilm thickness from the Z-stack image datasets was measured for individual samples (biological replicates) and averaged across each sample type (e.g., XR, SR, PA, MO) based on the signal detected in the SYTO60 channel, as SYTO60 stains cell biomass.

All analyses and plotting were done in RStudio v4.1.1 [97] with package “ggplot2” v3.4.2. All datasets were tested for normality and equal variance using the Shapiro-Wilk (base R) and Levene's (package “car” v3.1.2) tests, respectively. Datasets were not normally distributed and thereby a Kruskal-Wallis test (base R) was used when comparing more than two groups. A general expression was kruskal.test (Thickness ∼ Sample), however the quantitative variable and categorical variables changed depending on the dataset. A Dunn's post-hoc test was performed using package “FSA” v0.9.4 to consider multiple comparisons when the Kruskal-Wallis test was significant, with a Bonferroni correction. A p-value of 0.05 was used as a threshold for statistical significance (*p* < 0.05). Compact letter display for multiple comparisons was calculated using the R package “rcompanion” v2.4.35.

### 2.6 Biofilm matrix extraction

Biofilms were grown for 24 h in TSB medium on PC chips, followed by washing once in 1x PBS to remove planktonic cells and biofilm pellicles. Five biological replicates per condition were processed, each composed of five PC chips combined to extract sufficient biofilm matrix material for proteomics analysis (technical replicates). The biofilm was then resuspended in 5 ml of 1x PBS by shaking at 1000 rpm for 10 min. The biofilm matrix was extracted using the formaldehyde and NaOH extraction method previously described [38,39]. Briefly, 0.006 V formaldehyde 37 % (30 μl) was added to the biofilm samples and incubated for 1 h at 4 °C while mixing, followed by addition of 0.4 V NaOH 1 N (2 ml) and incubation for 3 h at 4 °C while mixing. Samples were then dialyzed using 3500 MWCO 22 mm SnakeSkin™ Dialysis Tubing (ThermoFisher Scientific) against 2.5 l Milli-Q water overnight at 4 °C with stirring. Protein content in dialyzed samples was quantified using the Bio-Rad Protein assay, a modified Bradford assay [40], before freezing with liquid nitrogen. Finally, the extracted biofilm matrix was lyophilized using an ALPHA 1–4 LDplus freeze dryer (Christ, VWR) and kept at −80 °C until sample preparation for proteomics.

For the proteomics sample preparation, digestion and mass spectrometry were conducted as described in Herschend et al. [13], with some modifications described in the supplementary methods.

### 2.7 Trimming of reference proteomes

The genomes of the MO, PA, SR, and XR are available online (NCBI) with IDs CP118897, CP118896, CP118898 and CP118899, respectively. Reference proteomes were based on the genomes. Each proteome was loaded into the statistical software environment R with SeqinR v3.31 package and *in silico* trypsin digested using the Cleaver v1.10.2 package. No missed cleavages were allowed. Resulting peptides were filtered for a minimum length of 7 amino acids, and were compared between all four reference proteomes for identical string matches. Any peptide found in two or more species was removed from all reference proteomes, by deleting the amino acid strings and concatenating the adjacent fragments. This resulted in trimmed reference proteomes with protein sequences containing only the trypsin-digested peptides unique to each species. The R script used for trimming reference proteomes can be found in the supplementary material.

### 2.8 Statistical analysis of matrix proteomics data and visualization

The raw data was analyzed using MaxQuant v1.6.10.43 [41] with the built-in Andromeda search engine [42]. A target decoy search allowed for a maximum of 1 % FDR on both peptide and protein levels and a minimum length of 7 amino acids per peptide. Quantification was performed using the label-free quantification (LFQ) algorithm [43] in MaxQuant with a minimum ratio count of 2 and applying the “match between runs” function. The Andromeda search engine was supplemented with the reference proteomes of MO, PA, SR, and XR. These genomes are available online (NCBI) with IDs CP118897, CP118896, CP118898 and CP118899, respectively. The raw data and results of the mass spectrometry proteomics analysis by MaxQuant have been deposited to the ProteomeXchange Consortium via the PRIDE [44] partner repository with the dataset identifier PXD057669.

Statistical analysis of proteomics data was performed with the Perseus software v1.6.15.0 [45]. The differential abundance of identified proteins was assessed using a modified version of Welch's *t*-test with an S0 parameter of 0.5 [46] and a permutation-based FDR cut-off of 0.05 and valid values in at least 60 % of the samples in both sample groups (minimum valid values = 3), depending on the comparison made. Prediction of subcellular localization of detected proteins was done using subcellular localization prediction software DeepLocPro 1.0 (https://services.healthtech.dtu.dk/services/DeepLocPro-1.0/) [47]. Visualization was done in RStudio v4.1.1 [48] and package “ggplot2” v3.4.2.

## 3 Results

### 3.1 Fluorescent lectin screening demonstrates the presence of various matrix glycans in multispecies biofilms

**Fig. 1:**
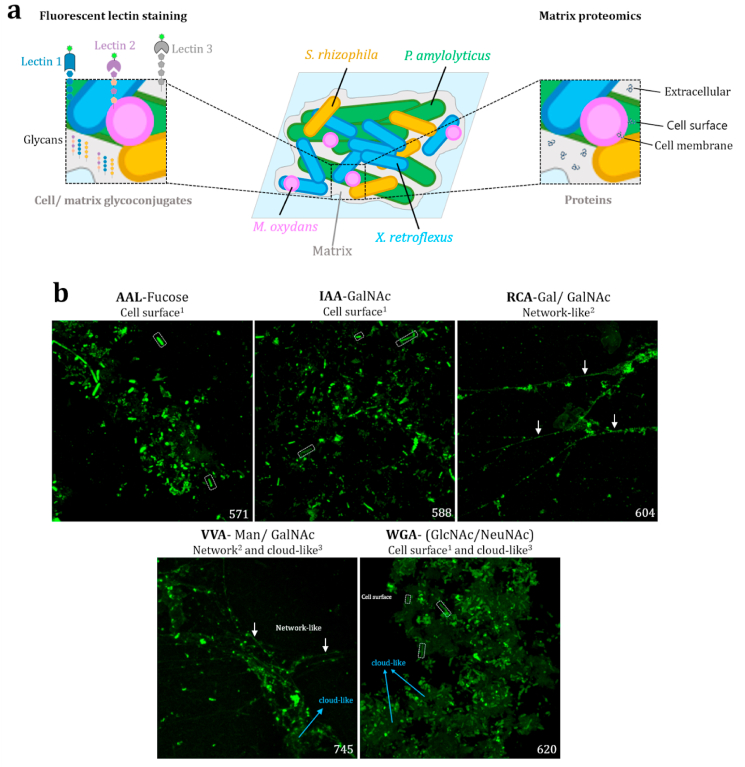
**Identification of matrix components in biofilms.** a) Experimental approach including i) fluorescent lectin screening of multispecies biofilms to visualize cell associated/matrix glyconconjugates; ii) Matrix proteomics to identify membrane, surface, or extracellular proteins with unique/differential abundance in mono- or multispecies biofilms. b) Lectins displaying strong binding to multispecies biofilms. The selected lectins, the glycan residues they bind, and the type of structures they stain, are indicated on top of the images. Some examples of cell surface binding (^1^) are highlighted as discontinued boxes, network-like structures (^2^) with white arrows, and cloud-like structures (^3^) with light blue arrows. PMT3 gain (green channel) is indicated at the bottom right corner of each picture. AAL: AAL- Alexa488. RCA: RCA-Fluorescein. VVA: VVA-FITC; WGA: WGA-FITC. IAA: IAA-Alexa488. Images are 123 × 123 μm and maximum intensity projection (MIP). (For interpretation of the references to colour in this figure legend, the reader is referred to the Web version of this article.)

To investigate how interspecies interactions influence the composition of the biofilm matrix, we initially focused on the glycoconjugates produced in multispecies biofilms. Given the complexity of biofilm communities, we hypothesized that the presence of multiple species would result in a more diverse array of glycoconjugates. To test this hypothesis, we employed fluorescent lectin binding analysis (FLBA) combined with confocal laser scanning microscopy (CLSM) to examine specific carbohydrate presence and interactions in the biofilm matrix (Fig. 1). Our screening involved 78 different fluorescent lectins binding to carbohydrates of various types. All lectins used in this study, the residues they bind, and their supplier are listed in Supplementary Table S1. Carbohydrate specificity of such lectins has been described in the literature as well as by their suppliers [[31], [37]].

After filtering the binding results of the screening by fluorescence signal [37], we identified five lectins that exhibited a strong fluorescent signal in multispecies biofilms: AAL, IAA, RCA, VVA, and WGA (Fig. 1b). AAL binds α-fucose-containing polymers, IAA and VVA bind N-acetylgalactosamine (GalNAc), RCA binds galactose and N-acetylgalactosamine (GalNAc) glycans, while WGA binds N-acetylglucosamine (GlcNAc) and N-acetylneuraminic acid (NeuNAc), a sialic acid [30,31,49,50]. The EPS glycoconjugates produced in multispecies biofilms, could be classified as cell-surface associated, network/filament-like, and cloud-like structures (Fig. 1b), depending on what type of structures they bind. Based on lectin specificity, cell-surface associated EPS glycoconjugates contained mainly fucose, amino sugars (GlcNAc and GalNAc), and sialic acid. Such structures were defined as surrounding the bacterial cells, with either strong or weak signal. Amino sugars and sialic acid dominated cloud-like EPS glycoconjugates, defined as a matrix encasing the cells displaying a weak fluorescent signal. Last, network-like structures were dominated by GalNAc and galactose (Fig. 1b). These structures resembled a mesh or web of thin polymers. Given that AAL and IAA both seem to stain cell surfaces, we employed IAA, RCA, VVA, and WGA for further investigation, representing lectins binding to different structures in multispecies biofilms.

### 3.2 Interspecies interactions between *X. retroflexus* and other community partners induce the production of matrix polymers

To determine whether interspecies interactions impacted the production of matrix glycans, we examined the binding patterns of lectins in both monospecies and multispecies biofilms. Given the known synergistic interaction between XR and PA in biofilm formation and co-localization [[14], [34]], co-culture biofilms of such strains were also analyzed. Additionally, biofilms were stained with SYTO60, a nucleic acid that stains cell biomass. The combined detection of cell biomass (SYTO60) and lectin signals allowed for a more comprehensive assessment of the spatial organization of cells and matrix glycoconjugates within the biofilm samples.

We opted for a qualitative approach to analyze glycans in the biofilm matrix due to the challenges associated with lectin fluorescence intensity-based quantification. Factors such as staining variability, photobleaching, and differences in imaging conditions can introduce inconsistencies, making absolute quantification unreliable [31,51,52]. Moreover, we observed that matrix structures varied not only between different lectins but also within the same lectin across biofilms of different species and their mixtures, further complicating quantitative comparisons. By focusing on spatial distribution, we provide a qualitative assessment of glycan localization.

However, to ensure reliable interpretations, we quantified biofilm thickness using the SYTO60 channel, allowing for standardized biofilm biomass assessment. Biofilm thickness did not significantly change among individual monospecies samples (Fig. S1). The dual-species biofilm of XR and PA tended to be thicker than monospecies biofilms and was significantly different from XR but not PA monospecies biofilm (Fig. S1). This suggest that biofilm architecture is highly influenced by PA. Thickness of multispecies biofilms was similar to that of dual-species biofilms but larger than single SR or MO biofilm. Additionally, we quantified biofilm thickness per lectin within each sample type, to assess whether different image sets could result in thickness changes. However, we observed no significant differences per sample type regardless of the lectin analyzed (Fig. S2, multiple comparison Kruskal-Wallis test). Thus, we proceeded to compare lectin binding and type of structures present in the different biofilm samples.

**Fig. 2:**
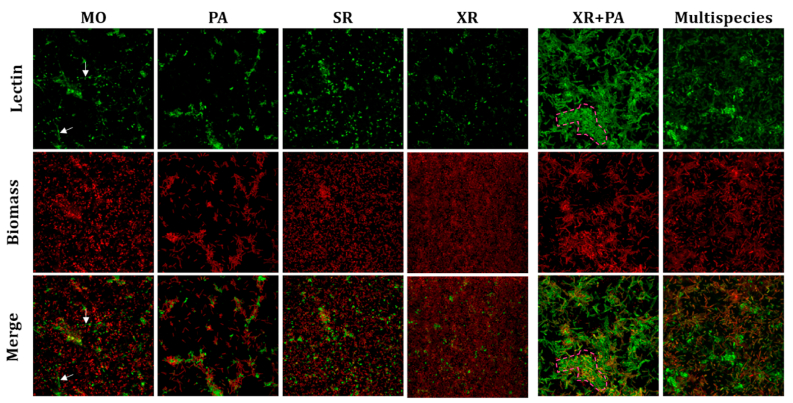
**Maximum intensity projections of mono-, dual-, and multispecies biofilms stained with lectin IAA and SYTO60**. MO: *M. oxydans*, PA: *P. amylolyticus*; SR: *S. rhizophila*, XR: *X. retroflexus*; XR + PA: co-culture of *X. retroflesus* and *P. amylolyticus*; Multispecies: all four species. 24-hour mono, dual- or multispecies biofilms were stained with the fluorescent lectin IAA-Alexa488 (top row) and SYTO60 (middle row) as cell biomass stain and imaged with a 63x water-immersion objective. Merged images in the bottom row show combined lectin and biomass channels. Examples of network-like structures are indicated with white triangles while raceme-like structures are depicted as discontinued pink shapes, respectively. Images are 123 × 123 μm. (For interpretation of the references to colour in this figure legend, the reader is referred to the Web version of this article.)

We found that IAA lectin bound all monospecies biofilms. However, it stained different types of structures (Fig. 2). In MO, IAA stained network-like and some cell-surface structures (as illustrated in Fig. 1b), while in SR and XR it mainly stained the cell surface. In PA biofilms, IAA binding was associated with cloud-like structures when cells aggregated in bigger clusters. Co-culture biofilms of XR and PA led to intense binding of IAA on PA cell surface —based on the larger cell size of PA— and branching formations of cell clusters and/or glycoconjugates, referred to as raceme-like structures—given their resemblance with a raceme inflorescence. This type of structure is likely influenced by interactions between the bacterial species PA and XR and their production of specific matrix glycoconjugates like GalNAc. Moreover, IAA binding was not as intense in multispecies as in PA + XR co-culture biofilms, though still observed more often than in monospecies biofilms.

**Fig. 3:**
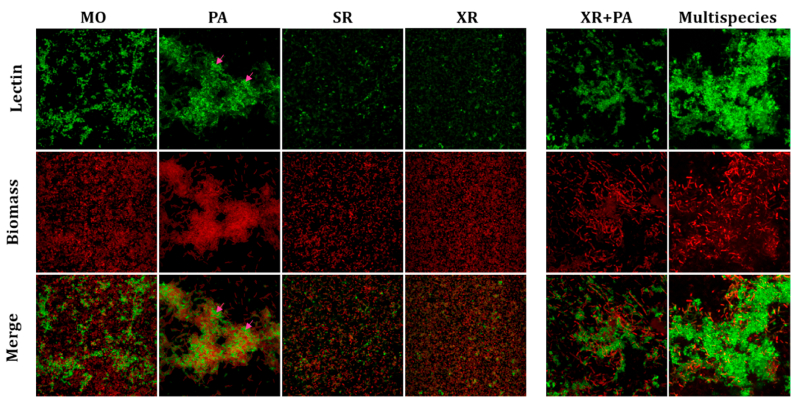
**Maximum intensity projections of mono-, dual-, and multispecies biofilms with lectin RCA and SYTO60**. MO: *M. oxydans*, PA: *P. amylolyticus*; SR: *S. rhizophila*, XR: *X. retroflexus*; XR + PA: co-culture of *X. retroflesus* and *P. amylolyticus*; Multispecies: all four species. 24-hour mono-, dual- or multispecies biofilms were stained with the fluorescent lectin RCA-Fluorescein (top row) and SYTO60 (middle row) as cell biomass stain and imaged with a 63x water-immersion objective. Pink arrows indicate examples of structures resembling adhesive scaffold. Merged images in the bottom row show combined lectin and biomass channels. Images are 123 × 123 μm. (For interpretation of the references to colour in this figure legend, the reader is referred to the Web version of this article.)

RCA lectin also bound differently in monospecies biofilms of the different strains (Fig. 3). Little binding was detected for SR and XR, but MO biofilms showed network-like RCA-bound structures. In contrast, PA showed distinct structures, characterized by intense fluorescent RCA lectin encasing PA cells, resembling an adhesive scaffold binding the cells together. Such matrix seemed associated with cells organized in large clusters. However, those structures weren't detected in XR + PA biofilms. Instead, diffuse, cloud-like structures (as illustrated in Fig. 1b) were detected, where the RCA signal appeared weaker. These results suggest a galactose/GalNAc polymer type shift in response to other species. Mainly, multispecies biofilms induced cloud-like structures encasing cluster-associated cells.

**Fig. 4:**
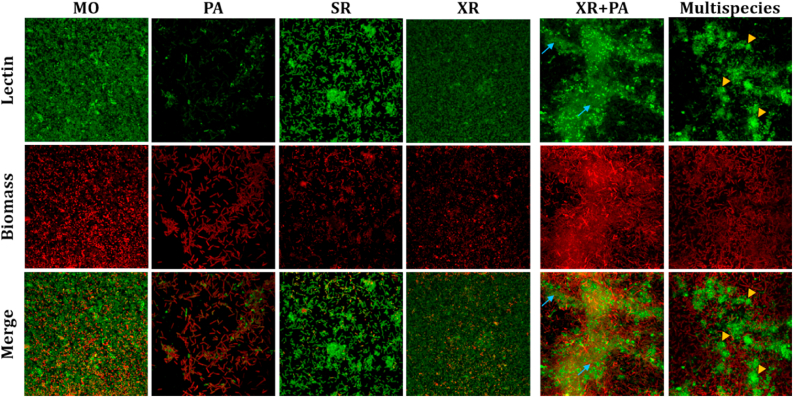
**Maximum intensity projections of mono-, dual-, and multispecies biofilms with lectin WGA and SYTO60**. MO: *M. oxydans*, PA: *P. amylolyticus*; SR: *S. rhizophila*, XR: *X. retroflexus*; XR + PA: co-culture of *X. retroflesus* and *P. amylolyticus*; Multispecies: all four species. 24-hour mono-, dual- or multispecies biofilms were stained with the fluorescent lectin WGA-FITC (top row) and SYTO60 (middle row) as cell biomass stain and imaged with a 63x water-immersion objective. Examples of cloud-like structures are indicated by blue arrows while globule-like structures are indicated by yellow arrows. Merged images in the bottom row show combined lectin and biomass channels. Images are 123 × 123 μm. (For interpretation of the references to colour in this figure legend, the reader is referred to the Web version of this article.)

WGA lectin seemed to stain the cell surface in monospecies biofilms of MO, SR, and XR, while little signal was detected around PA cells (Fig. 4). However, XR + PA biofilms produced sialic acid/GlcNAC cloud-like WGA-binding structures (Fig. 4, blue arrows), encasing cell clusters. Interestingly, multispecies biofilms displayed globule-like structures, characterized by prominent WGA lectin signal and shapes resembling globules, suggesting a high density of glycoconjugates (Fig. 4, yellow arrows). Since such structures were not detected in XR + PA biofilms, we hypothesize that either MO or SR are responsible for the induction of such structures in the presence of XR and/or PA.

Last, VVA lectin showed low binding to monospecies biofilms of all species, and cultivation in dual species (XR + PA) or multispecies only resulted in a discrete induction of lectin binding (Fig. S3).

In conclusion, FLBA allowed the identification of different types of glycan residues present in mono-, dual-, or multispecies biofilms and indicated shifts in glycoconjugate structures in response to other species.

### 3.3 Co-localization of *X. retroflexus* and fucose/N-Galactosamine polymers in multispecies biofilms

RCA lectin showed a distinctive staining pattern in biofilms in comparison to other lectins analyzed. It revealed distinct glycan structures in monospecies of PA and MO, with maximal binding in multispecies biofilms. Consequently, we aimed to further investigate the production of galactose and GalNac polymers in response to interspecies interactions. We used a fluorescent version of XR, which was chromosomally tagged with *gfp*, in both mono- and multispecies biofilms. The rationale behind this choice was that XR has previously been shown to be abundant in multispecies biofilms, effective at forming biofilms on its own, and to interact with other members of the consortium [12,10,14,53].

**Fig. 5:**
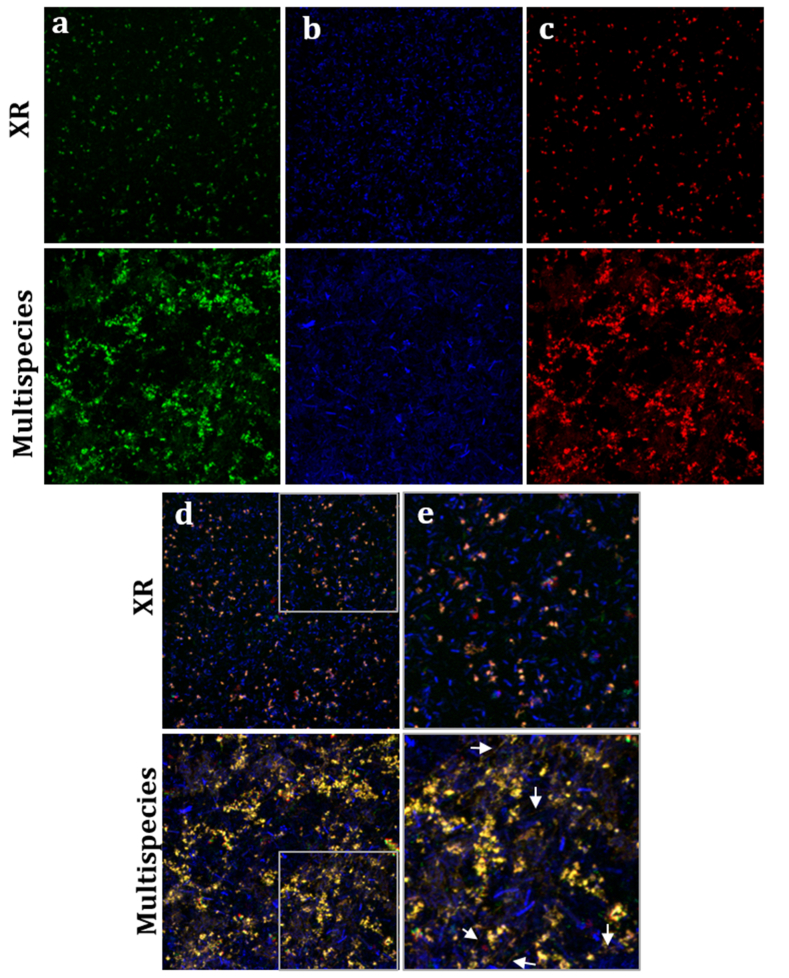
**Maximum intensity projections of *X. retroflexus* mono- and multispecies biofilms with lectin RCA and SYTO60**. Biofilms of *X. retroflexus* (XR) tagged with *gfp* (a, green) in mono- or multispecies 24-h biofilms were stained with SYTO60 (b, blue) as cell biomass stain, and the fluorescent lectin RCA-Rhodamine (c, red), and imaged with a 63x water-immersion objective. Merged images in the fourth column show combined GFP, cell biomass, and RCA lectin channels (d and e). Images are 123 × 123 μm. e) Regions of interest highlighted from merged images (3x magnification). White arrows indicate examples of network-like structures. Images represent 41 × 41 μm. (For interpretation of the references to colour in this figure legend, the reader is referred to the Web version of this article.)

In this case, we employed a Rhodamine-conjugated RCA lectin to complement the green, fluorescent signal from XR-*gfp* cells. Monospecies biofilms of XR-*gfp*, stained with SYTO60 and RCA-Rhodamine, corroborated our previous findings, showing minimal RCA binding (Fig. 5).

In contrast, multispecies biofilms containing XR-*gfp* demonstrated a high abundance of XR in the presence of other species, revealing cloud- and network-like structures. The overlapping red and green fluorescence signals suggested two possibilities: i) XR uniquely synthesizes galactose/GalNAc-containing polymers in the presence of other species, or ii) XR co-localizes with another species responsible for producing these polymers. Network-like polymer structures were detected exclusively in MO monospecies biofilms but not in XR biofilms (Fig. 3). These results suggest that XR and MO likely co-localize in multispecies biofilms.

### 3.4 Matrix proteomics as a tool to identify matrix proteins in response to interspecies interactions

**Fig. 6:**
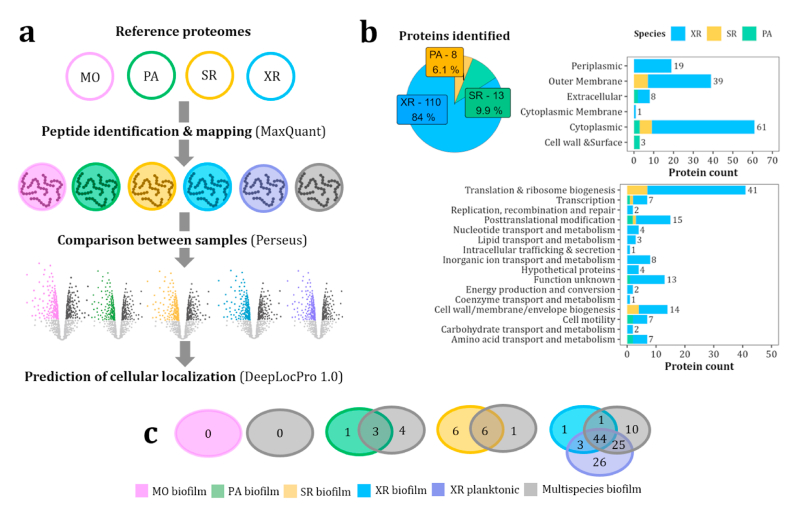
**Matrix proteomics of mono- and multispecies samples using trimmed reference proteomes.** MO: *M. oxydans*, PA: *P. amylolyticus*; SR: *S. rhizophila*, XR: *X. retroflexus*; XR + PA: co-culture of *X. retroflesus* and *P. amylolyticus*; Multispecies: all four species. All samples are biofilms, except for XR planktonic**. a)** Workflow of matrix proteomics analysis. **b)** Proteins identified per species, cellular localization and role category (COG). Cellular localization of identified proteins was predicted using the tool DeepLocPro 1.0. [47]. Role categories correspond to COG categories. **c)** Venn diagrams of mono- and multispecies samples per species. Proteins detected in each condition are indicated for each comparison.

To further investigate the composition of the biofilm matrix, we applied matrix proteomics to identify matrix components that were differentially or uniquely abundant in monospecies versus multispecies biofilms (Fig. 6a). This approach provided a comprehensive understanding of the protein components associated with biofilm formation and species interactions. We aimed at identifying extracellular, surface- or membrane-associated proteins that may play critical roles in multispecies biofilm structure and function (Fig. 1), providing insights into how microbial communities adapt and collaborate within a shared extracellular matrix. Additionally, we included XR planktonic samples, to evaluate the validity of the extraction technique as well as potential differences in protein abundance between planktonic and biofilm lifestyle in this species.

Biofilms were detached from the PC chips, matrix extracted, dialyzed, proteolytically cleaved, fractionated, and analyzed by mass spectrometry (MS) based proteomics according to materials and methods. We used both reference proteomes of XR and SR to map peptides to specific proteins of SR, XR, and multispecies samples, given that these species are phylogenetically close. Initial analysis of the MS data from the four-species biofilm resulted in 199 proteins identified. XR was the most abundant species based on the number of proteins identified (n = 125, Fig. S4a), followed by SR (n = 20). On the contrary MO and PA were of minor abundance based on the fact that 0 and 9 proteins were identified, respectively (Fig. S4a).

However, 23 % of all proteins identified could not be resolved between SR and XR (n = 45), suggesting substantial peptide overlap between these species. Moreover, we detected cross-mapping in monospecies samples, that is, mapping to other species than that on the sample (Fig. S4b). In MO samples 36 proteins were identified, however, none of them mapped to MO reference proteome. Also, proteins identified in monospecies samples mapped to other species. Strikingly, no proteins were identified in SR samples. However, 20 proteins identified in other conditions mapped to the SR reference proteome (Figs. S4a–b). Interestingly, all proteins detected in PA mapped exclusively to its reference proteome, suggesting a lower degree of peptide overlap with other species.

To circumvent the issue of overlapping proteins between different species, especially SR and XR, we compared each reference proteome with each other, to identify unique shared peptides. Peptides common to species pairs were trimmed from the reference proteomes, and trimmed reference proteomes generated for performing the peptide search and protein identification (Fig. S5a). Application of the trimmed reference proteomes led to a substantial reduction in the number of peptides common for XR and SR (9.1 and 9.6 %, respectively), in contrast to MO and PA (0.05 and 0.1 %, Fig. S5b and Table S2). Analysis with the trimmed reference proteomes resulted in 131 proteins identified, 84 % mapping to XR proteome (Fig. 6b and Table S3). Thus, despite the use of trimmed reference proteomes, the highest number of proteins was still identified in XR. The rest of proteins mapped to SR and PA proteomes (9.9 and 6.1 %), while no proteins were identified for MO. This was also consistent with the analysis of untrimmed reference proteomes. Trimming of reference proteomes efficiently eliminated cross-mapping, as all proteins in monospecies samples mapped to their respective species reference proteomes (Table S3).

We also predicted the cellular localization of identified proteins using DeepLocPro 1.0 tool [47]. From all proteins identified, 47 % were cytoplasmic, 30 % outer membrane, 14 % periplasmic, 6 % extracellular and 2 % associated to the cell wall or surface. Only one protein was predicted to be located in the cytoplasmic membrane (Fig. 6b). Moreover, the most represented role categories were translation and ribosome biogenesis (31 %), posttranslational modification and cell wall/membrane biogenesis, accounting for 52 % of all proteins identified (Fig. 6b). We also identified seven proteins involved in cell motility and eight in inorganic ion transport and metabolism.

Comparisons of identified proteins within species (multispecies vs. monospecies) also evidenced differential abundance of different proteins in response to species interactions (Fig. 6c). Three proteins showed differential abundance for PA (Table S4), 6 for SR (Table S4) and 44 shared for all XR samples (Table S5 and Fig. S6). Moreover, we also identified multiple proteins with unique abundance in the different samples (Table S6).

Interestingly, we found two S-layer homology domain-containing proteins (SLPs) in PA samples (PWP87_29955 and PWP87_29960), with significantly higher abundance in multispecies biofilms than monospecies. Bacterial S-layer proteins have been associated with adherence to various substrates, aggregation and coaggregation with yeasts and other bacteria [54]. Both identified proteins are predicted to be associated with the cell wall (Table S4). Another SLP was only detected in multispecies samples (PWP87_04380), associated with cell motility (Table S4) as well as an extracellular peroxiredoxin and a thioredoxin.

In SR, we found four proteins in the cell wall/membrane/envelope biogenesis category, two detected in both multispecies and SR monospecies samples and two only detected in SR monospecies biofilms (Table S4). These included a Ton-B dependent receptor and two outer membrane beta-barel proteins (PWP89_04390 and PWP89_05845).

For XR, we found 6 extracellular proteins, 4 flagellins, a hypothetical protein and an autotransporter (Table S3). Moreover, multiple proteins showed differential abundance in the different samples (Fig. S6 and Table S5). Additionally, we identified five TonB-dependent receptors belonging to the Inorganic ion transport and metabolism category (Table S3). We found that one of them, PWP_07845, was significantly more abundant in multispecies biofilms, compared to either XR biofilm samples or XR planktonic.

In summary, the use of trimmed reference proteomes effectively reduced cross-mapping, allowing for more precise protein identification and localization within the multispecies biofilm matrix. Unique proteins in PA and XR, such as S-layer homology proteins and TonB-dependent receptors, highlighted key adaptive features in multispecies biofilms.

### 3.5 Flagellum and cell wall proteins as potential central social components in multispecies biofilms

Analyzing proteomics samples with trimmed reference proteomes revealed multiple proteins involved in cell motility and cell wall/membrane biogenesis with unique or differential abundance in the different samples and species (Table S3).

**Fig. 7:**
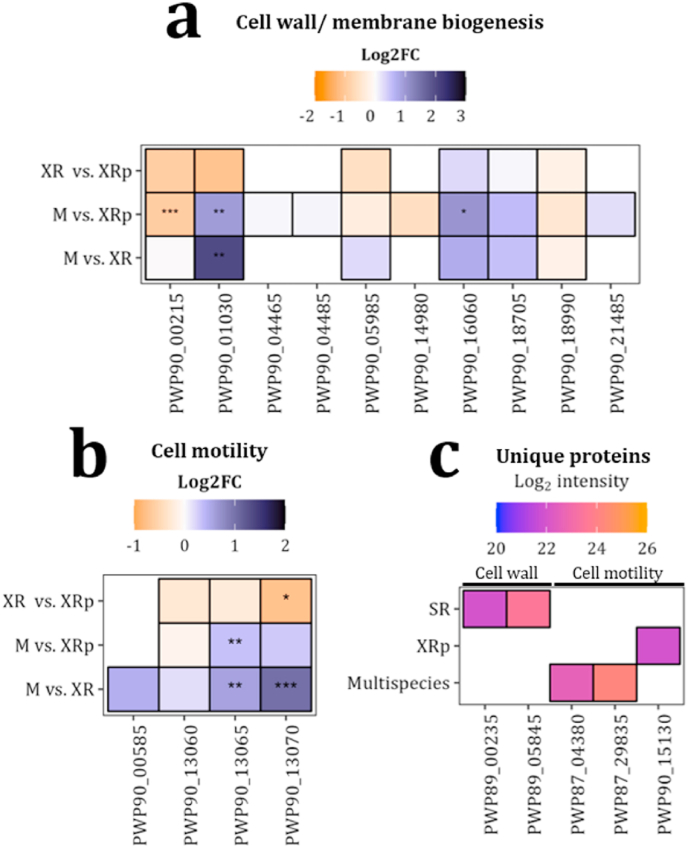
**Proteins identified in the cell wall and motility categories. a)** Heatmap of proteins identified in XR for cell wall/membrane biogenesis. Color indicates abundance difference, expressed as Log_2_ fold change (Log2FC) of multispecies vs. XR biofilm (M vs. XR), multispecies vs. XR planktonic (M vs. XRp), and XR biofilm vs. XR planktonic (XR vs. XRp). Positive and negative values indicate higher protein abundance in the first or second group, respectively. Asterisks indicate the level of significance of the comparison (Welch's *t*-test; ∗: *p* < 0.05; ∗∗: *p* < 0.01; ∗∗∗: *p* < 0.001). b) Heatmap of XR proteins identified in the cell motility category, illustrated as in panel a). **c)** Unique proteins from the cell wall/membrane biogenesis or cell motility detected only in specific conditions. SR: detected in SR monospecies biofilms; XRp: detected in XR monospecies planktonic samples; Multispecies: detected in multispecies biofilm samples. Color indicates Log2 transformed protein intensity. (For interpretation of the references to colour in this figure legend, the reader is referred to the Web version of this article.)

First, we further analyzed those proteins showing differential abundance in both categories. Fourteen proteins were identified corresponding to the cell wall/membrane biogenesis, 4 from SR and 10 from XR. We further analyzed XR proteins since they showed differential abundance in different conditions. These included two Ax21 family proteins (PWP90_00215 and PWP90_01030), two outer membrane beta-barrel proteins (PWP90_04465 and PWP90_05985), and two peptidases (PWP90_21485 and PWP90_16060), among others (Fig. 7a and Table S3). PWP_01030 showed significant higher abundance in multispecies samples compared to XR biofilm or planktonic samples while PWP90_00215 showed significant higher abundance in XR planktonic samples compared to multispecies biofilms, and the opposite for PWP90_16060. This suggests that not only species interactions, but also bacterial lifestyle could influence abundance of such Ax21 family proteins.

Regarding cell motility proteins with differential abundance, we identified flagellin proteins common to all XR samples (XR, XR planktonic, and multispecies), including several flagellins with higher abundance in multispecies when compared to monospecies biofilms (Table S5 and Fig. 7b). PWP_13065 and PWP_13070 were significantly more abundant in multispecies vs. monospecies XR biofilms, while more abundant in XR planktonic vs. XR biofilm samples. Furthermore, the flagellin PWP90_13065 showed significant higher abundance in multispecies biofilms compared to XR monospecies planktonic or biofilm samples. The other two flagellins detected (PWP90_13065 and PWP_00585) also showed higher abundance in multispecies vs XR monospecies, though not significant. These results suggest that motility is enhanced in response to interspecies interactions (multispecies biofilms > XR monospecies biofilms) but also to lifestyle (planktonic > biofilm).

In relation to proteins in such categories with unique detection, we could identify other flagellin proteins in XR and PA but only under specific conditions (Fig. 7c and Table S6). In PA*,* the flagellin Hag (PWP87_29835) was identified only in multispecies biofilms, suggesting that abundance of this protein depends on interspecies interactions (Fig. 7c and Table S6). Similarly, PWP87_04380, a SLP, was also detected exclusively in multispecies biofilms. This protein is annotated in the cell motility category.

The differential flagellin detection patterns may suggest that motility within biofilms may be induced or reduced in response to other species. Alternatively, we hypothesize that flagellin could serve as a matrix scaffold for other community members.

## 4 Discussion

EPS consists of various macromolecules, making it difficult to characterize its composition accurately and at high resolution using single analytical or staining methods. This complexity pertains to the EPS as a whole and applies to specific groups of components, such as glycans and proteins. Many studies have employed Concanavalin A (ConA) or wheat germ agglutinin (WGA) lectins to visualize glycans in biofilms due to their broader specificity [31,32]. However, glycans are among the most intricate macromolecules found in nature, not only because of the diversity of their fundamental building blocks but also because of the variety of bonds that can form at each carbon.

The FLBA applied in this study evidences the increased diversity of glycans observed in our multispecies biofilms, with fucose, GalNAc, galactose, GlcNAc, and sialic acid-containing polymers identified through the lectins AAL, IAA, RCA, VVA, and WGA. Such findings align with the understanding that biofilm matrices are highly heterogeneous, and composition and structure can differ significantly based on the type of microorganisms present, local shear stress, nutrient or substrate availability, and the surrounding environment [1]. This highlights the broader ecological implications of interspecies interactions in biofilm formation, as these interactions contribute to emergent properties such as enhanced protection, structural resilience and metabolic cooperation, which may impact biofilm persistence in natural and engineered environments.

We also provide evidence that interspecies interactions within multispecies biofilms significantly influence matrix glycan production and spatial organization. In dual-species biofilms of XR and PA, we observed strong IAA-binding to GalNAc-containing glycans, especially in raceme-like structures around PA cells, but also matrix-like structures stained with WGA. These species seem to cooperate in co-culture, evidenced by increased biofilm biomass, pH stabilization, and co-localization on root surfaces [14,18,34]. Moreover, the results of the present study show that matrix glycan production is induced in co-culture biofilms of both species and suggest that both species may mutually benefit from matrix glycan production. Other researchers have previously proven that EPS synthesis and spatial arrangement vary between monospecies and multispecies microbial communities [55]. For instance, co-cultures of *S. mutans* with other oral bacteria are known to upregulate glucan synthesis, remodeling, and binding, thus contributing to enhanced biofilm stability and structural complexity in response to interspecies interactions [22]. These findings further emphasize that interspecies interactions shape biofilm matrix composition, possibly affecting nutrient exchange, physical stability, and protection from environmental stressors, which could have implications for medical, industrial, and environmental biofilm management strategies.

The reduced IAA binding in multispecies biofilms compared to XR + PA biofilms (Fig. 2) suggests that while GalNAc-containing polymers are still produced, the presence of additional species such as MO and SR might lead to competition or alternative pathways of glycan production. MO has previously been characterized as a low-abundance species in the multispecies consortium used in this study. Despite its minor biomass contribution, MO significantly influenced spatial organization within the multispecies biofilm, enhanced the growth of poorer biofilm formers, and altered community composition, emphasizing its role in stabilizing the biofilm's structure and function [19]. Interestingly, our results indicate MO as a key species for specific production of different matrix glycoconjugates, including galactose/GalNAc network-like structures, evidenced by the FLBA of MO monospecies biofilms (Fig. 2, Fig. 3). Such network-like structures were also observed in multispecies biofilms and co-localized with XR, unlike monospecies biofilms of XR (Fig. 5). This is consistent with previous studies on the spatial organization of such strains, where XR and MO showed local intermixing in four-species biofilms, as opposed to dual species, and exhibited the highest degree of intermixing in mature biofilms compared to other species pairs [53]. In addition, MO failed to stably reside in three-species biofilms whenever XR was absent, supporting mutual facilitation between these species during the maturation of the four-species biofilm [19]. Our results underscore the challenge of defining a universal matrix composition, as it varies with species contributions, metabolic interactions, and environmental conditions.

Previous studies have demonstrated that biofilm matrix composition varies significantly depending on species interactions and environmental factors. For example, Flemming & Wingender [4] emphasize that biofilm matrices are highly dynamic and cannot be universally characterized due to their dependence on local conditions. Similarly, Bridier et al. [56] showed that biofilm spatial organization is constantly adapting to environmental pressures, making broad generalizations challenging. The presence of specific microbial interactions can also lead to emergent properties that are not predictable from single-species biofilms, as observed in co-adhesion studies of *Streptococcus mutans* and *Candida albicans* [57]. It is therefore highly relevant to analyze biofilm matrix regulation in a context-dependent manner to account for the variability observed in different microbial communities.

Label-free quantification of proteins from the extracted matrix allowed us to identify matrix proteins from all four species. Even though few proteins were identified for PA, three of them corresponded to S-layer proteins (SLPs). SLPs are key players in bacterial biofilm-related traits by promoting surface adhesion, providing structural integrity, aiding in immune evasion, and offering protection against antimicrobial agents [54]. Wong et al. described an SLP serving as a public good matrix exopolymer in mixed species biofilms, facilitating recruitment of other bacteria and enabling syntrophic relationships [58]. In *Paenibacillus alvei*, Janesh et al. associated an SLP with swarming motility and biofilm formation [59]. Remarkably, our results evidenced a significantly higher abundance of two SLPs in multispecies compared to monospecies biofilms and another with unique expression in multispecies, belonging to the cell motility category. Furthermore, we identified a peroxidase uniquely present in multispecies biofilms. This peroxidase has an 89 % identity to AphA of *Bacillus subtilis*, a biofilm-specific peroxidase regulated by the major sporulation and biofilm regulator Spo0A [60]. Most studies describing the involvement of bacterial peroxidases in biofilms are associated with pathogenic bacteria, suggesting a role in virulence [61]. However, our results are in accordance with previous research, reporting the role of multispecies biofilms in promoting resistance to oxidative stress [62]. We also identified multiple TonB-dependent receptors (TBDR) in SR and XR. TBDRs are primarily known for their role in transporting nutrients, such as iron-siderophores and vitamins, across the outer membrane of Gram-negative bacteria [63]. They have also been linked to biofilm formation in different bacteria [[64], [65], [66]]. Different enzymes were also detected, including multiple peptidases and peptidyl-prolyl cis/trans isomerases (PPIases). Peptidades are involved in peptidoglycan (PG) turnover during vegetative growth, cell division, motility, or biofilm formation, including PG endopeptidases [67]. On the other hand, bacterial PPIases are involved in metabolism, virulence, and multiple stress responses, but also in biofilm and pellicle formation in different bacteria [[68], [69], [70]].

Proteomics analysis further evidenced differential or unique abundance of multiple flagellin proteins in different species (Fig. 7). Flagellin proteins were more abundant in multispecies biofilms—except for PWP90_13070- in XR planktonic samples, suggesting a beneficial role in these conditions. Jung et al. reported the involvement of flagellin-homologous proteins (FHPs) in biofilm maturation and interaction with exopolysaccharides in the matrix [71]. Ozer et al. found that flagella are synthetized throughout the life cycle of *P. aeruginosa,* but lack of flagella results in weaker biofilm compared to the wild-type strain, suggesting a structural role of flagella within the biofilm [72] Moreover, in *Geobacter* sp., the flagellum serves as a biofilm matrix scaffold, stabilizing biofilm and facilitating electron diffusion within the biofilm [73]. Given that no other proteins related to the flagellar assembly machinery were identified in our analysis, we speculate that flagellin proteins might exert a structural function in the matrix, especially in multispecies biofilms, conceivably as a scaffold.

We also identified three Ax21 family proteins, two in XR and one in SR. Ax21 family proteins play a crucial role in bacterial signaling and plant defense responses. These proteins are secreted by certain pathogenic bacteria, such as *Xanthomonas oryzae* and *Stenotrophomonas maltophilia*, where they function as signaling peptides involved in quorum sensing. Mutations in Ax21 in *S. malthophila* lead to reduced motility and reduced biofilm formation [74,75]. Our results showed significant higher abundance of PWP90_01030 in multispecies biofilms compared to XR biofilms or planktonic samples, suggesting also a role in biofilm in the presence of other species.

Despite the identification of extracellular or membrane-associated proteins, our matrix proteomics results showed a dominance of cytoplasmic proteins in the pool of proteins identified from mono- and multispecies biofilms, suggesting leakage of cytoplasmic proteins due to cell lysis or matrix extraction. Over the past decade, it has been described that many proteins can have one or more unique functions, known as protein moonlighting [76,77]. Different studies have reported that the biofilm matrix of Staphylococci is primarily composed of cytoplasmic proteins reversely associated with the cell surface [24,78]. Additionally, secreted proteins can also contribute to the composition and formation of biofilm in Staphylococci [79]. A number of the cytosolic proteins found in the *P. aeruginosa* biofilm matrix have also been reported to have moonlighting activities involved in host cell interactions in *P. aeruginosa* and other bacteria [77]. Thus, moonlighting via an unknown exportation pathway may explain the presence of such proteins on the cell surface and in the extracellular environment in the samples analyzed in the present study.

In summary, our research emphasizes the complex arrangement of glycans and proteins in the biofilm matrix. The identification of a range of glycan-specific binding patterns seems to be influenced by the presence of key species like MO and XR. At the same time, the detection of matrix proteins, including surface-layer proteins or flagellins, underscores their critical roles in biofilm stability and structural integrity. These findings offer a deeper understanding of how interspecies interactions regulate matrix production and suggest that the biofilm environment promotes cooperative behavior, likely enhancing the resilience and functionality of microbial communities. Moreover, the dynamic and species-specific nature of biofilm matrix composition presents challenges for broad generalizations, requiring context-dependent analyses to fully understand biofilm behavior. Recognizing these complexities may aid in developing targeted approaches for biofilm control and utilization in industrial, clinical, and environmental applications.

## CRediT authorship contribution statement

**Cristina I. Amador:** Writing – review & editing, Writing – original draft, Visualization, Validation, Supervision, Project administration, Methodology, Investigation, Formal analysis, Data curation, Conceptualization. **Henriette L. Røder:** Writing – review & editing, Validation, Methodology, Investigation, Funding acquisition, Formal analysis, Data curation, Conceptualization. **Jakob Herschend:** Writing – review & editing, Methodology, Data curation. **Thomas R. Neu:** Writing – review & editing, Methodology, Investigation. **Mette Burmølle:** Writing – review & editing, Supervision, Project administration, Funding acquisition, Conceptualization.

## Declaration of competing interest

The authors declare the following financial interests/personal relationships which may be considered as potential competing interests:

Mette Burmolle reports financial support was provided by European Research Council. Mette Burmølle reports financial support was provided by Office of Naval Research. Henriette Lyng Røder reports financial support was provided by Villum Foundation. If there are other authors, they declare that they have no known competing financial interests or personal relationships that could have appeared to influence the work reported in this paper.

## Data Availability

The mass spectrometry proteomics raw data and results from analysis by MaxQuant have been deposited to the ProteomeXchange Consortium via the PRIDE [45] partner repository with the dataset identifier PXD057669 (https://www.ebi.ac.uk/pride/archive). The data is currently private and can only be accessed with a single reviewer account that has been created.
