# Supplementary material for: Decoding the impact of interspecies interactions on biofilm matrix components
Source: Biofilm. 2025 Mar 14;9:100271. doi: 10.1016/j.bioflm.2025.100271 (PMC11985002; doi:10.1016/j.bioflm.2025.100271)
Supplement: Multimedia component 1 [file mmc1.docx]

**Supplementary material**

Decoding the impact of interspecies interactions on biofilm matrix components

**This file includes:**

- Supplementary methods
- Supplementary figures : 6 supporting figures
- Supplementary R-scripts : 1 supporting R-script
- Supplementary tables can be found in the file “Amador et al 2024_Supplementary tables.xlsx”, containing 6 supporting tables

**Supplementary Materials and Methods**

**Mass Spectrometry analysis**

For each sample, peptides were loaded onto a 2cm C18 trap column (ThermoFisher 164705), connected in-line to a 15cm C18 reverse-phase analytical column (Thermo EasySpray ES803) using 100% Buffer A (0.1% Formic acid in water) at 750bar, using the Thermo EasyLC 1000 HPLC system, and the column oven operating at 45 °C. Peptides were eluted over a 200 minute gradient ranging from 10 to 60% of 80% acetonitrile, 0.1% formic acid at 250 nl/min, and the Q-Exactive instrument (Thermo Fisher Scientific) was run in a DD-MS2 top10 method. Full MS spectra were collected at a resolution of 70,000 with an AGC target of 3×106 or maximum injection time of 20 ms and a scan range of 300–1750 m/z. The MS2 spectra were obtained at a resolution of 17,500, with an AGC target value of 1×106 or maximum injection time of 60 ms, a normalised collision energy of 25 and an intensity threshold of 1.7e4. Dynamic exclusion was set to 60 s, and ions with a charge state <2 or unknown were excluded. MS performance was verified for consistency by running complex cell lysate quality control standards, and chromatography was monitored to check for reproducibility.

**Supplementary figures**


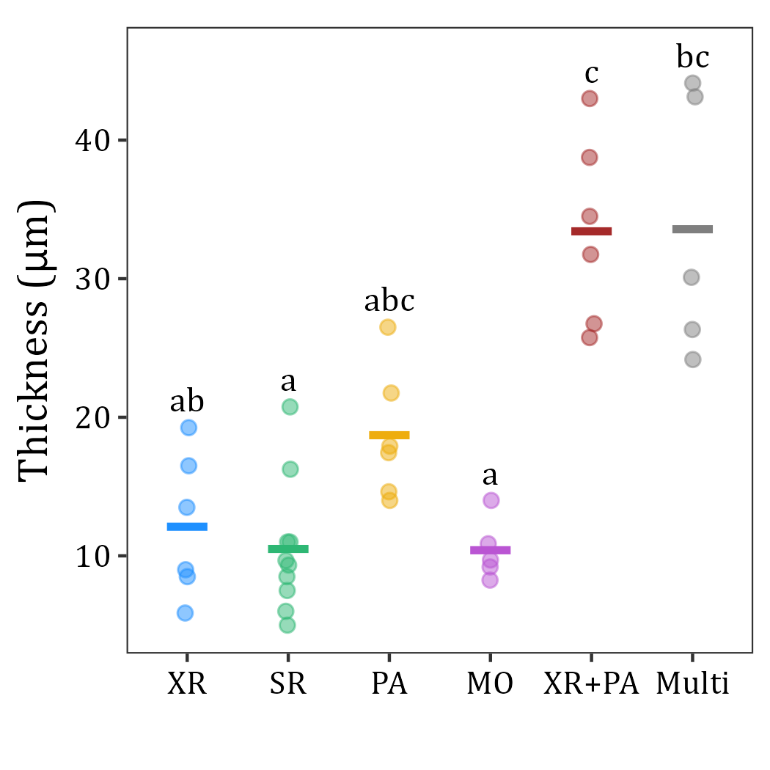


**Figure S1. Biofilm thickness of mono-, dual- and multispecies samples**. Average thickness, calculated from SYTO60 channel (cell biomass stain), is shown as horizontal crossbars. Each individual point represent a biological replicate and coupon. Dissimilar letters indicate significantly different p-values of a multiple comparison Dunn test (*p* < 0.05).


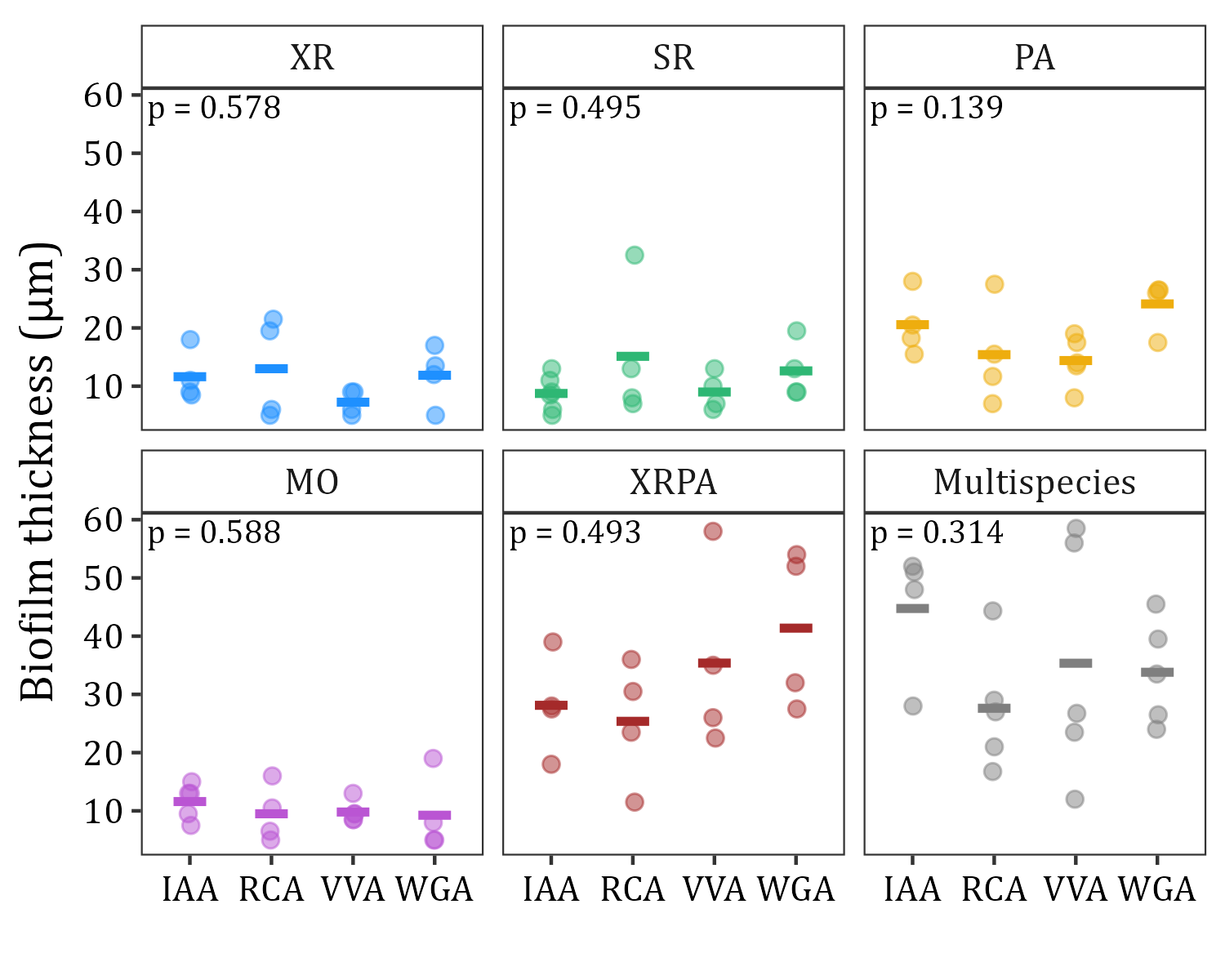


**Figure S2. Biofilm thickness of mono-, dual- and multispecies samples per sample type and lectin**. Average thickness, calculated from SYTO60 channel (cell biomass stain), is shown as horizontal crossbars. Each individual point represent a biological replicate and coupon. “*p*” indicates the p-value of a Kruskal-Wallis test (*p* < 0.05) for each sample type. None of the samples present significant differences for biofilm thickness regardless of the lectin used.


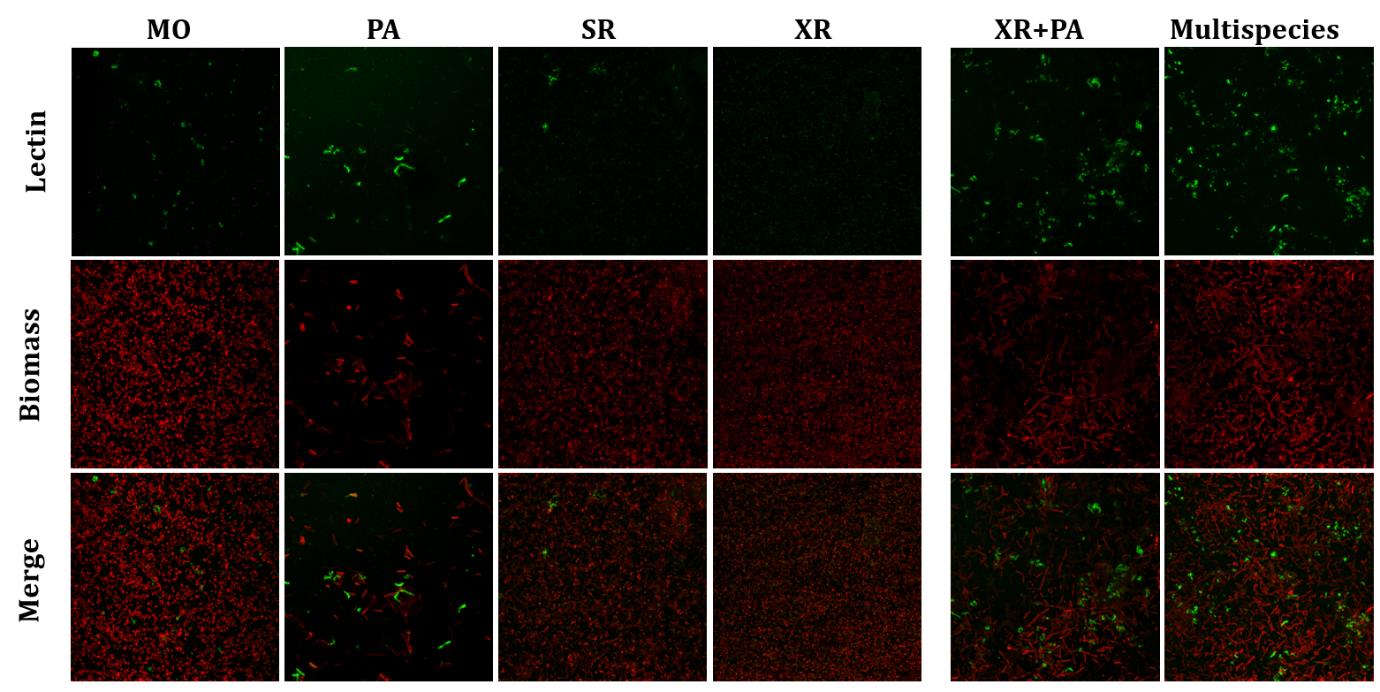


**Figure S3. Maximum intensity projections of mono-, dual-, and multispecies biofilms with lectin VVA and SYTO60**. 24-hour biofilms were stained with VVA-FITC (top row) and Syto60 (middle row) as cell biomass stain and imaged with a 63x water-immersed objective. Merged images in the bottom row show combined lectin and biomass channels. Images are 123x123 µm.

**
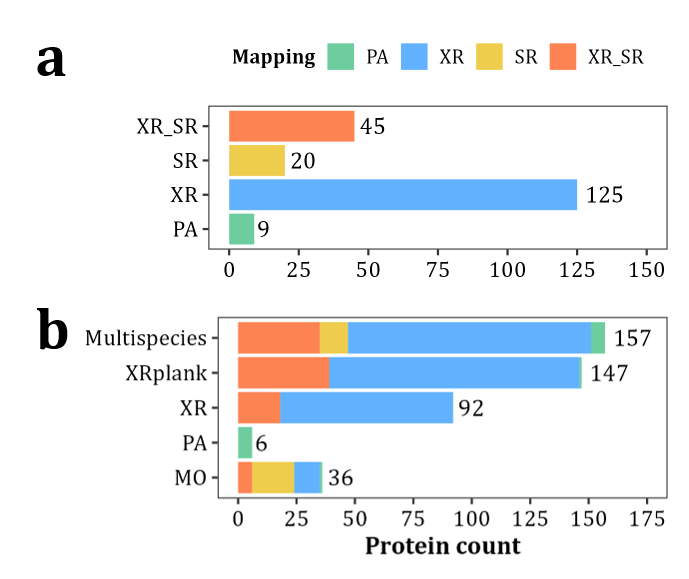
**

**Figure S4. Proteins identified using untrimmed reference proteomes and their mapping to the different species.** a) Protein count of identified proteins based on their mapping to reference proteomes. XR_SR indicate proteins where the analysis could not distinguish between XR or SR species. b) Protein count by sample type: monospecies MO, PA, XR, XR planktonic or multispecies samples. Mapping to reference proteomes is shown as in a). No proteins were identified in MO samples.

**
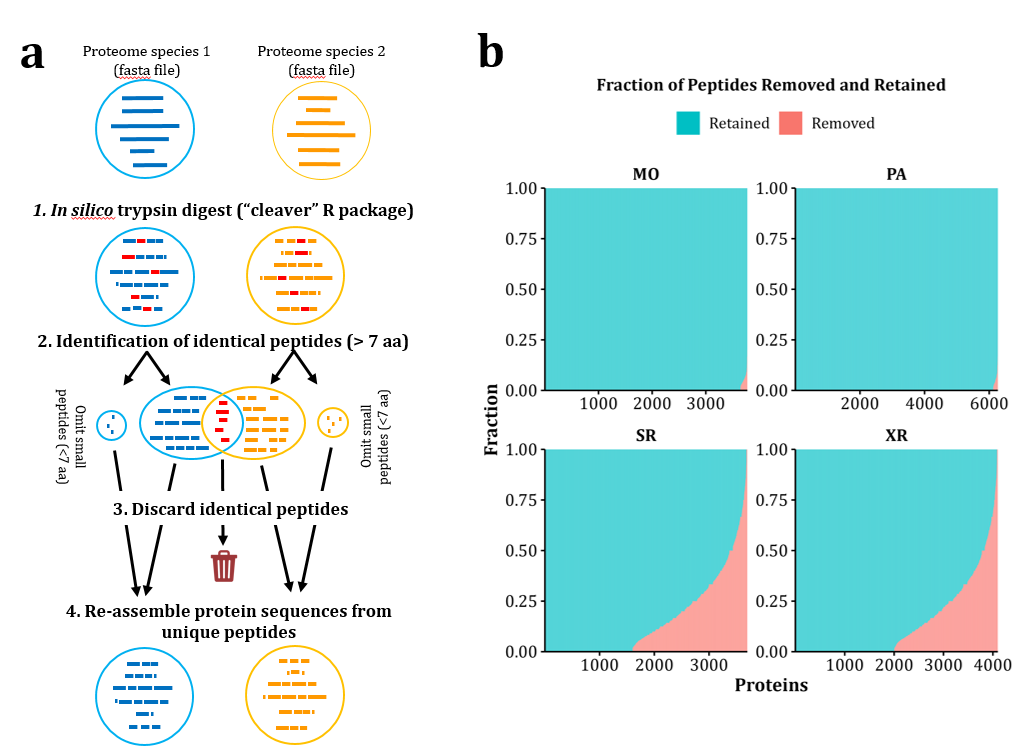
**

**Figure S5. Workflow for trimming of reference proteomes.** The reference proteomes were in silico digested with trypsin. Resulting peptides were binned according to their length. Peptides >7 amino acids (aa) were compared to peptide sequences from all the other reference proteomes and identical peptides from each reference proteome (in red), discarded. The remaining peptides >7 aa and peptides <7 were then re-assembled into protein sequences, now only containing peptides that are unique to that species.


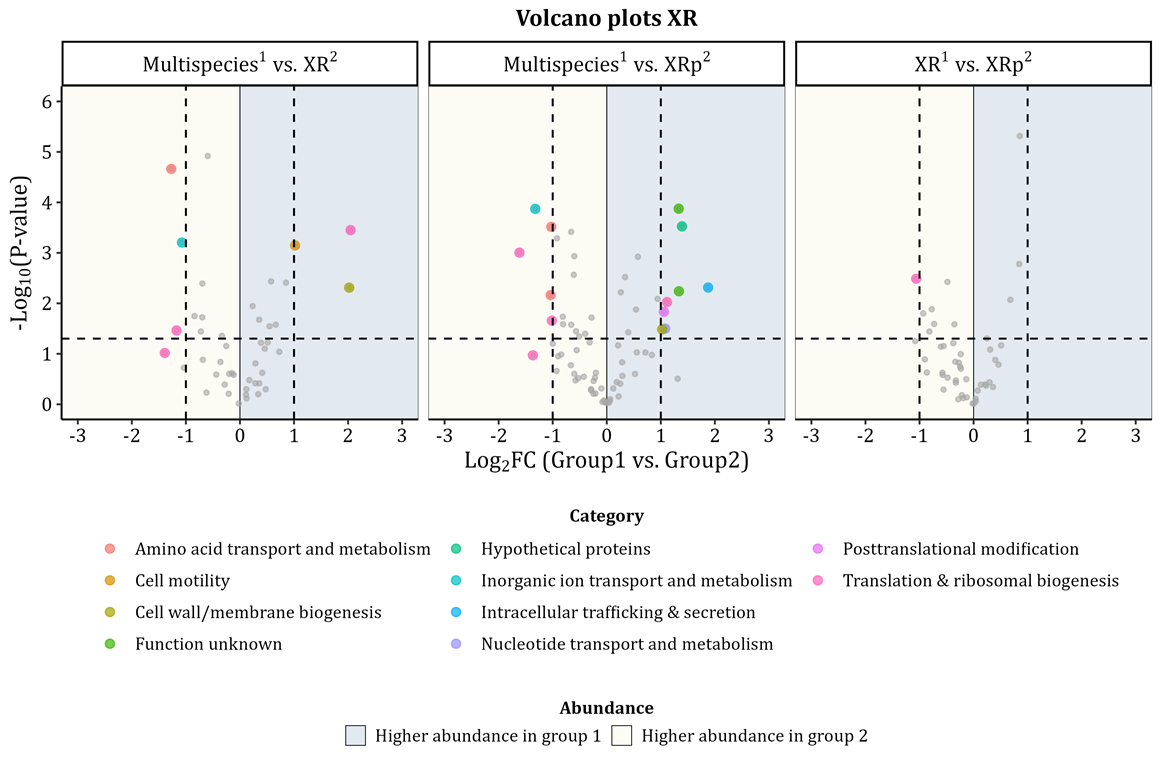


**Figure S6. *X. retroflexus* proteins with differential abundance in XR planktonic and XR and multispecies biofilm samples, identified using trimmed reference proteomes.** Proteins with significant changes in abundances were identified using a modified Welch’ s t-test with a S0 constant of 0.5 and valid values in at least 3 out 5 biological replicates per sample type. A false discovery rate (FDR) correction was also applied with a significance threshold of 0.05. Volcano plots show the –Log10 p-values (y axis) versus abundance difference between the first and second sample groups, expressed as Log2 transformed fold change (Log_2_FC). Each panel shows a different comparison between sample groups: Multispecies vs. monospecies XR biofilms( left), Multispecies biofilms vs. monospecies XR planktonic (middle), and monospecies XR biofilm vs. XR planktonic (right). Proteins with significant p-values and/ or Log2FC change (-1<Log_2_FC>1) are shown in colors according to the role categories (COG). Grey points denote proteins with non-significant p-values and/ or Log_2_FC between -1 and 1. The blue an ivory rectangles per panel indicate proteins with higher abundance in the first (^1^) or second group (^2^).

**Supplementary R-script**

Supplementary R-script 1: Preparation of trimmed reference proteomes

# ------------------------------------- General information and used Packages ----------------------------------------

## updated October 2024, to work on any number of fasta files

# Install packages used in script

require("BiocManager")

install.packages("BiocManager")

BiocManager::install("cleaver", force = TRUE)

install.packages("seqinr")

install.packages("seqinr", type = "source")

# Load packages used in script

library(cleaver)

library(seqinr)

library(ggplot2)

library(reshape2)

library(writexl)

library(dplyr)

library(extrafont)

loadfonts()

# ----------------------------------------- Load fasta files, and do in silico digest ---------------------------------------

filesall <- c("SM_converted.faa", "SR_converted.faa", "MO_converted.faa", "PA_converted.faa") # write file names

## Read the Proteome fasta files.

## 'seqtype' argument describes whether it is DNA or AA.

FASTAall <- lapply(filesall, function(f) {

read.fasta(file = f, seqtype = "AA", as.string = TRUE)

})

# [specific to our case]

# Example:

# from: "PWP90_05320"

# to: ""PWP90"

species.names <- sapply(FASTAall, function(fa) {

sub("\\_[0-9]+$", "", names(fa)[1])

})

# Perform trypsin digestion on all sequences within each FASTA file

DIGESTSall <- lapply(FASTAall, function(fa) {

cleave(unlist(fa), enzym = "trypsin")

})

# Assign names to the FASTA and DIGESTS lists

names(FASTAall) <- names(DIGESTSall) <- names(filesall) <- species.names

# Perform the count of peptides per species

total_peptides_per_species <- sapply(DIGESTSall, function(peptides) {

total_peptides <- sum(sapply(peptides, length))

return(total_peptides)

})

# Convert to data frame for better readability (optional)

result_df <- data.frame(Species = names(total_peptides_per_species),

TotalPeptides = total_peptides_per_species)

# View the results

print(result_df)

########################################################################

#---------------Simplify the data for faster analysis --------------------------

# The list of fragments is turned into a vector, fragments shorter than 7 are

# removed and the peptides are grouped according to their length. The peptides

# need to be grouped into their peptide length so that you only compare

# peptides of eg. 11aa with peptides from the other organism that are also

# 11aa. These steps significantly reduce the time for the analysis.

preparePeptidomes <- function(x, min.length) {

names(x) <- paste0(names(x), "_")

x <- unlist(x) # Turn the list into a vector

x <- x[nchar(x) >= 7] # Remove fragments shorter than 7

x <- split(x, nchar(x)) # Group the peptides by length

return(x)

}

#########################################################################

#---------Find peptides that are present in more than one species --------------

# Find peptides that are present in both sequences

intersectPeptidomes <- function(PLIST, ...) {

# input must already be digested (use the output from 'cleave')

PLIST <- lapply(PLIST, preparePeptidomes, ...)

# Get all the combinations to compare

pairs <- combn(length(PLIST), 2)

# Now for each combination of proteomes...

shared <- apply(pairs, 2, function(x) {

P1 <- PLIST[[ x[1] ]] # first proteome to compare

P2 <- PLIST[[ x[2] ]] # second proteome to compare with

message("Intersecting proteomes ", names(PLIST)[x[1]], " and ", names(PLIST)[x[2]])

# For each of the peptide lengths in the first proteome (7mers, 8mers, 9mers, etc...)

tmp <- lapply(names(P1), function(l) {

if(l %in% names(P2)) {

# Now this function also returns the organism and protein ids,

# so it is faster to find these peptides in the fasta files afterwards

# Compare all peptides in 1 and 2

mat <- outer(X = P1[[l]], Y = P2[[l]], FUN = `==`)

# Get the organism/protein name for those that were shared

shared_rows <- apply(mat, 1, any)

shared_cols <- apply(mat, 2, any)

shared_P1 <- P1[[l]][shared_rows]

shared_P2 <- P2[[l]][shared_cols]

# Return a vector of the shared peps

c(shared_P1, shared_P2)

}

# if no overlap, returns empty char vector

# if no peps in P2 of that length, returns NULL.

# Doesn't really matter once you use 'unlist' on the result

})

unlist(tmp)

})

unlist(shared)

}

# Intersect peptidomes

shared_peps_all <- intersectPeptidomes(DIGESTSall, min.length = 7)

#Count the number of shared peptides

if (length(shared_peps_all) == 0) {

cat("No shared peptides found.\n")

} else {

cat("Number of shared peptides:", length(shared_peps_all), "\n")

cat("Contents of shared peptides:\n")

print(shared_peps_all)

}

####################################################################

#----------------------------Reformat the results ------------------------------

# Remove the trailing number, which was introduced by R to keep track of multiple

#proteins from same species

# Example:

# from: "PWP90_05320_18"

# to: ""PWP90_05320"

prots <- sub("_[0-9]+$", "", names(shared_peps_all))

#Make table of protein names and peptides, one row per peptide.

sp_mat <- cbind(protein = prots, peptide = unname(shared_peps_all))

# Get the species name, by removing the protein id from the end

species <- sub("\\_[0-9]+$", "", prots)

# Make list of tables, one table per species

sp_list <- split.data.frame(sp_mat, species)

# ------------ Remove shared peps and create new fasta files -------------------

NEW_FASTAall <- FASTAall

# For each proteome/FASTA

for (i in species.names) {

cat("\nProcessing peptides in FASTA: ", i, "\n")

# for each line in the file...

for (j in 1:nrow(sp_list[[i]])) {

# current line, which consists of:

# 1) protein (the protein name)

# 2) peptide (the aa sequence)

prot <- sp_list[[i]][j,]["protein"]

pep <- sp_list[[i]][j,]["peptide"]

# In the relevant proteome, take the relevant protein, and substitute the peptide sequence with nothing ("")

NEW_FASTAall[[i]][[prot]] <- gsub(pep, "", NEW_FASTAall[[i]][[prot]])

if(j%%1000 == 0) cat("Processed ", j, " peptides in FASTA: ", i, "\n")

}

}

############################################################################

# ------------------Write new fasta files to disk ------------------------------

for (i in names(NEW_FASTAall)) {

# make new file name from the old one

new.file <- sub("(\\.\\w+)$", ".UNIQUE\\1", filesall[i])

write.fasta(NEW_FASTAall[[i]],

names = names(NEW_FASTAall[[i]]),

as.string = TRUE,

file.out = new.file)

message("Saved new FASTA file for species ", i, " as ", new.file)

}

################################################################################

# ---------- Compare peptide sets before and after -----------------------------

# print table of non-unique peptides

write.table(do.call(rbind, lapply(names(sp_list), function(species) cbind(species, sp_list[[species]]))),

file = "table-of-removed-peptides.tsv", quote = FALSE, sep = "\t", row.names = FALSE)

# For each proteome/FASTA

countPeps <- lapply(setNames(species.names, species.names), function(i) {

prot_list <- split(sp_list[[i]][,"peptide"], sp_list[[i]][,"protein"])

# for protein in that proteome...

t(sapply(names(DIGESTSall[[i]]), function(j) {

# what's the number of digested peps in total for this protein? (only those 7 aas or longer!)

n.total <- sum( nchar(unique(DIGESTSall[[i]][[j]])) >= 7 )

# if the protein is not in the list of redundant peptides

if(! j %in% names(prot_list)) {

return(c(before = n.total, Removed = 0, Retained = n.total))

}

# If it is, on the other hand, count the number of redundant peps in this protein

n.removed <- length(unique(prot_list[[j]]))

return(c(before = n.total, Removed = n.removed, Retained = n.total - n.removed))

}))

})

countPeps <- lapply(countPeps, function(i) {

cbind(i, "percent.removed" = i[,"Removed"] / i[,"before"])

})

for (i in names(countPeps)) {

j <- sub("_.*", "", i)

write.table(countPeps[[i]], file = paste0(j, "_count-peptides.tsv"), col.names = NA, quote = FALSE, sep = "\t")

}

pdf("Retained-peptides-xy.pdf", width = 5, height = 5)

for (i in names(countPeps)) {

x <- countPeps[[i]]

plot(x[,"before"]+1, xlab = "Peptides per protein (+1)", x[,"Retained"]+1, ylab = "Retained peptides (+1)", las = 1, main = i, log = "xy", pch = 16, col = "#00000020") # log scale

}

species_titles <- c("PWP90" = "XR", "PWP89" = "SR", "PWP88" = "MO", "PWP87" = "PA")

dev.off()

pdf("Fraction-peptides-removed_3.pdf", width = 5, height = 4)

for (i in names(countPeps)) {

x <- countPeps[[i]]

ord <- order(x[,"percent.removed"])

levels <- unique(rownames(x)[ord])

tmp <- melt(x[,2:3])

tmp$Var1 <- factor(tmp$Var1, levels = levels)

tmp$Var1 <- as.numeric(tmp$Var1)

tmp$Var2 <- factor(tmp$Var2, levels = c("Retained", "Removed"))

colnames(tmp) <- c("Proteins", "Peptides", "Fraction")

title <- species_titles[i]# Retrieve the title for each species based on the species_titles vector

# Create the plot with the specified title

p <- ggplot(tmp, aes(x = Proteins, y = Fraction, fill = Peptides)) +

geom_bar(width = 1, stat = "identity", position = "fill") +

scale_fill_manual(name = "", labels = c("Retained", "Removed"),

values = c("#00bfc4", "#F8766D"))+

scale_x_continuous(expand = c(0,0)) +

scale_y_continuous(expand = c(0,0)) +

ggtitle(title)

print(p)

}

dev.off()

#-----------------------------------------------------End---------------------------------------------------------------------------
